# A Hybrid Genetic-Neural System for Predicting Protein Secondary Structure

**DOI:** 10.1186/1471-2105-6-S4-S3

**Published:** 2005-12-01

**Authors:** Giuliano Armano, Gianmaria Mancosu, Luciano Milanesi, Alessandro Orro, Massimiliano Saba, Eloisa Vargiu

**Affiliations:** 1 Dept. of Electrical and Electronic Engineering, University of Cagliari Piazza d'Armi, I-09123 Cagliari, Italy; 2 Shardna Life Sciences, Piazza Deffenu 4, I-09121 Cagliari, Italy; 3 Institute for Biomedical Technologies, National Research Council, Via Fratelli Cervi 93, I-20090 Segrate (MI), Italy

## Abstract

**Background:**

Due to the strict relation between protein function and structure, the prediction of protein 3D-structure has become one of the most important tasks in bioinformatics and proteomics. In fact, notwithstanding the increase of experimental data on protein structures available in public databases, the gap between known sequences and known tertiary structures is constantly increasing. The need for automatic methods has brought the development of several prediction and modelling tools, but a general methodology able to solve the problem has not yet been devised, and most methodologies concentrate on the simplified task of predicting secondary structure.

**Results:**

In this paper we concentrate on the problem of predicting secondary structures by adopting a technology based on multiple experts. The system performs an overall processing based on two main steps: first, a "sequence-to-structure" prediction is enforced by resorting to a population of hybrid (genetic-neural) experts, and then a "structure-to-structure" prediction is performed by resorting to an artificial neural network. Experiments, performed on sequences taken from well-known protein databases, allowed to reach an accuracy of about 76%, which is comparable to those obtained by state-of-the-art predictors.

**Conclusion:**

The adoption of a hybrid technique, which encompasses genetic and neural technologies, has demonstrated to be a promising approach in the task of protein secondary structure prediction.

## Background

The strict relationship between protein structure and function, together with the increasing gap between the number of sequences and known structures in bioinformatics databases, has lead to the need of automatic protein structure prediction systems. Unfortunately, protein structure prediction is a very difficult task. Chemical properties of aminoacids and complex interactions between different parts of the same protein and/or the protein and the surrounding environment should be taken into account for an accurate folding reconstruction. On the other hand, protein structure can be simplified by considering only the secondary structure, i.e. the local conformation which each residue belongs to, usually represented as: alpha-helix (*α*), beta-sheet (*β*), and random-coil (*c*). Although the knowledge about secondary structure is less informative than the tertiary one, an accurate secondary prediction can help in building the complete protein folding [1,2]. Furthermore, it can be a useful starting point to determine the protein function. In fact, most active sites are associated to particular conformations (or combinations) of secondary structures (motifs), more conserved during the evolution than the corresponding primary sequence.

Artificial neural networks (ANNs) are the most widely-used technology for predicting secondary structures. Typically, the input required to predict the secondary structure of a target residue is extracted from a windows of aminoacids centered on the residue itself, whereas three (i.e., *α*, *β*, and *c*) or eight (i.e., H, E, G, S, T, B, I, and ".") outputs represent the relevant structural classes. The prediction accuracy is commonly measured by the percent of residues correctly predicted versus the overall number of residues in the dataset (*Q*_*3*_). It is worth pointing out in advance that the typical output encoding adopts three values, and that the mapping between eight- to three-class encoding is not unique. In this paper, we adopted the "default" mapping proposed in [3], where α←{*H*, *G*, *I*}, β←{*E*, *B*}, and c represents everything else. The *Q*_*3 *_index is evaluated in accordance with this choice.

There are a variety of secondary structure prediction methods proposed in the literature. Early prediction methods were based on statistics headed at evaluating, for each amino acid, the likelihood of belonging to a given secondary structure [4]. The main drawback of these techniques is that, typically, no contextual information is taken into account, whereas nowadays it is well known that secondary structures are determined by chemical bonds that hold between spatially-close residues. A second generation of methods exhibits better performance by exploiting protein databases, as well as statistic information about amino acid subsequences. In this case, a limited window of aminoacids (e.g., 11 continuous residues) is taken into account, centered around the residue to be predicted. Several methods exist in this category, which may be classified according to (i) the underlying approach, e.g., statistical information [5], graph-theory [6], multivariate statistics [7], (ii) the kind of information actually taken into account, e.g., physico-chemical properties [8], sequence patterns [9], and (iii) the adopted technique, e.g., k-Nearest Neighbors [10], Artificial Neural Networks (ANNs) [11].

The most significant innovation introduced in prediction systems was the exploitation of long-range and evolutionary information contained in multiple alignments. The underlying motivation is that active regions of homologous sequences will typically adopt the same local structure, irrespective of local sequence variations. PHD [12] is one of the first ANN-based methods that make use of evolutionary information to perform secondary structure prediction. In particular, after searching similar sequences using BLASTP [13], ClustalW [14] is invoked to identify which residues can actually be substituted without compromising the functionality of the target sequence. To predict the secondary structure of the target sequence, the multiple alignment produced by ClustalW is given as input to a multi layer ANN. The first layer outputs a sequence-to-structure prediction which is sent to a further ANN layer that performs a structure-to-structure prediction aimed at refining it.

Further improvements are obtained with both more accurate multiple alignment strategies and more powerful neural network structures. For instance, PSI-PRED [15] exploits the position-specific scoring matrix (called "profile") built during a preprocessing performed by PSI-BLAST [16]. This approach outperforms PHD thanks to the PSI-BLAST ability of detecting distant homologies. In more recent work [3,17], recurrent ANNs (RANNs) are exploited to capture long-range interactions. The actual system that embodies such capabilities, i.e., SSPRO [18], is characterized by: (a) PSI-BLAST profiles for encoding inputs, (ii) bidirectional RANNs, and (iii) a predictor based on ensembles of RANNs. Further information about protein secondary structure prediction can be found, for instance, in [19].

## Methods

In this paper a system that resorts to multiple experts for dealing with the problem of predicting secondary structures is presented, whose performances are comparable to those obtained by other state-of-the-art predictors. The system, called MASSP3 (MultiAgent Secondary Structure Predictor with Post-Processing), performs an overall processing based on two main steps: first, a "sequence-to-structure" prediction is performed, by resorting to a population of hybrid genetic-neural experts, and then a "structure-to-structure" prediction is performed, by resorting to a feedforward ANN.

### Sequence-to-Structure Prediction

In this subsection the module that has been devised to perform the first step, which stems from the one proposed in [20], will be briefly described -focusing on the internal details that characterize an expert and on the behavior of the overall population of experts.

### Experts' "Internals"

**Figure 1:**
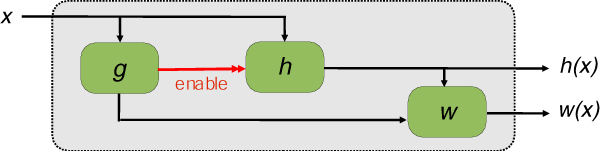
The micro-architecture of an expert.

In its basic form, each expert *E *can be represented by a triple <*g, h, w*> (as depicted in Figure 1), where: (i) *g *is a function used to select inputs according to the value of some relevant features, (ii) *h *is an embedded predictor whose activation depends on *g(x)*, and (iii) *w *is a weighting function used to perform output combination. In particular, the output of *E *coincides with *h(x) *for any selected input, otherwise it is not defined. In the case E contributes to the final prediction (together with other experts), its output is modulated by the value *w(x) *of its weighting function, which represents the expert confidence (i.e., its strength) about its own prediction.

In the proposed system, the guard g of an expert is implemented by a "genetic" classifier able to match inputs according to a set of selected features deemed relevant for the given application. Matching is "flexible", meaning that, for any given input *x*, *g(x) *∊ [0, 1], thus allowing experts to show different "ranges of authority" on *x*. Moreover, an expert can participate to the output combination activity only when the matching with *x *returns a value greater than a given threshold *μ*. In so doing, experts do not have complete visibility of the input space, i.e., they operate on different regions with "soft" boundaries.

As for the embedded predictor *h*, it typically consists of a feedforward ANN trained and activated on the inputs acknowledged by the corresponding guard. Each embedded predictor has three outputs corresponding to *α*, *β*, and *c*, all normalized in [0, 1], to facilitate output combination. The value *w(x)*, ranging over the interval [0, 1], depends on the expert fitness, on the result of the match, i.e. on *g(x)*, and on the reliability of the prediction made by the embedded predictor. The reliability *r *of a prediction is evaluated as the difference between the two highest values, as proposed in [12]. In the current implementation of the system, the weighting function of an expert *E *is: *w*_*E*_(*x*) = *f*_E_*g_*E*_(*x*)*r_*E*_(*x*).

In the task of predicting secondary structures, genetic guards are entrusted with processing some biological features, normalized in [0, 1], deemed relevant for the given task. This makes it possible to split the input space, with the goal of simplifying the training of neural classifiers. The corresponding features are reported in Table 1. For each feature, the operation actually performed on the current window *r *of residues, together with the underlying conjecture, are reported. The "AAindex" database [21] has been used for retrieving relevant information about residues, e.g., hydrophobicity, polarity, charge, and volume.

It is relatively easy to fulfil the constraint that each feature must be limited in [0, 1], although the specific technique used to quantitatively evaluate a feature is strictly dependant on the feature itself. For instance, feature 1 is obtained by performing a matching – which may include a preliminary shift between the actual window (representing residues according to their property of being hydrophobic or not) and a prototypical "target" window in which hydrophobicity fulfils the hypothesis about periodicity. In this case, the normalization in [0, 1] is included as part of the matching activity. In other cases, e.g., feature 4, the normalization is obtained by filtering with a suitable sigmoid the difference between the value of charge averaged on the given window and the corresponding expected value.

In so doing, for each given position to be predicted, a vector *m *of values in the range [0, 1] is first evaluated, and then supplied as input to the guard of each expert in the population. Each guard may accept or not the input depending on an embedded genetic pattern e, which is a string in {0, 1,#}. In particular, the i-th component of a genetic pattern being 0 (1) forces the corresponding feature to be close to 0 (1), whereas the dont-care symbol (i.e., "#") allows the corresponding feature to be disregarded. In practice, the i-th component of e controls the evaluation of the corresponding input features, so that only non-"#" features are actually taken into account. Hence, *H*_*g*_≠ φ being the set of all non-"#" indexes in *e*, *g(x) *can be defined, according to the Minkowski's *L*_∞ _distance metrics, as:



### Dealing with a Population of Experts

**Figure 2:**
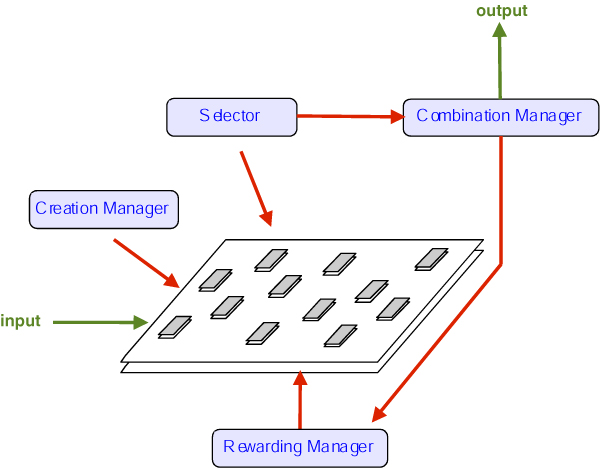
The architecture of the module that performs the sequence-to-structure processing.

The overall population of experts is dealt with according to the typical guidelines that hold for evolutionary environments. As shown in Figure 2, experts interact with: (i) a selector, (ii) a combination manager, (iii) a rewarding manager, and (iv) a creation manager. The selector is devoted to collect all experts whose guard covers the given input, thus forming the match set *M*. The combination manager is entrusted with combining the outputs of experts belonging to *M*, so that a suitable voting policy can be enforced on them. The main task of the rewarding manager is to force all experts in *M *to update their internal parameters, according to the reward obtained by the external environment. The creation manager is responsible for creating experts, when needed.

In the current implementation of the system, the training activity encompasses two steps: (i) discover a population of guards aimed at soft partitioning the input space, and (ii) train the embedded predictors of the resulting population.

In the first step, experts are generated and selected concentrating only on their soft-partitioning capability, rather than on the precision of the system. In fact, in this phase, embedded predictors play a secondary role, their training being deferred to the second step. Until then, embedded predictors output only the statistics (in terms of percent of *α*, *β*, and *c*) computed on the subset of inputs acknowledged by their guard (which, in this phase, does not depend on the input x being processed).

**Figure 3:**
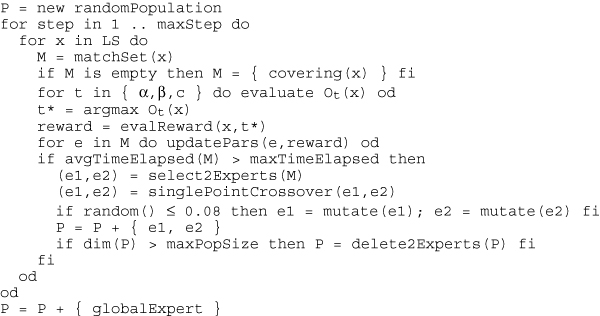
The pseudo-code of the "soft-partitioning" algorithm (LS = learning set).

Figure 3 illustrates the underlying training activity. The system starts with an initial population of experts equipped with randomly-generated guards. The population undergoes several training epochs, until the maximum number of epochs (i.e., 40) has been reached. At each epoch, the whole learning set is processed.

Triggered by an input *x *of class *t*_*x*_∊ {α,β,*c*}, the match set *M *is built, consisting of all experts whose guards cover *x*. When *M *is empty a new expert is created, whose genetic guard is able to cover the current input. In this case, an embedded pattern (built upon the alphabet {0, 1,#} able to match *x *is generated using a greedy algorithm driven by the goal of maximizing the percent of inputs that share the class *t*_*x*_within the inputs covered by the guard itself. The overall prediction (in terms of *α*, *β*, and *c*) is evaluated by averaging for each class, the output of all experts in *M *and then selecting the class with highest value. Let us note that the contribution of each expert *E *is weighted according to *w*_*E*_.

After that, the reward from the environment is calculated and the parameters that characterize each expert in *M*, including fitness, are updated according to the guidelines of Wilson's XCS systems [22]. If the average time elapsed from the last mating operation (evaluated on the experts in *M*) exceeds a predefined threshold, a pair of experts is selected in *M *according to a probability that increases with fitness. These experts are used to generate a new pair of experts using genetic operators (single-point crossover and possibly mutation, with probability 0.08), to be inserted in the population. Single-point crossover and mutation operate on the embedded patterns of the parents. If the population limit has been reached, a pair of experts to be deleted is selected. The probability of deleting a given expert is proportional to the estimated average size of the match sets in which it occurred, and increases for experts with low fitness.

It is worth pointing out that, at the end of the first step, a globally-scoped expert (i.e., equipped with a guard whose embedded pattern contains only "#") is inserted in the population, to guarantee that the input space is completely covered in any case. Upon completion of the first step, no further creation of experts is performed.

In the second step, the focus moves to embedded predictors, which are actually multi-layer perceptrons (MLPs) trained using the backpropagation algorithm on the subset of inputs acknowledged by their corresponding guard. In the current implementation of the system, all MLPs are trained in parallel, until a convergence criterion is satisfied or the maximum number of epochs (i.e., 20) has been reached. In its basic form, MLP training is performed by enforcing backpropagation on the set of inputs acknowledged by the corresponding guard. A further training technique has also been experimented, explicitly devised for this specific application: Given an expert consisting of a guard g and its embedded predictor h, h is trained on the whole training set in the first five epochs. The visibility of the training set is restricted on the subsequent epochs according to the inputs filtered in by g. In this way, a mixed training technique is performed, whose rationale lies in the fact that experts must find a suitable trade-of between the need of enforcing diversity (by specializing themselves on a relevant subset of the input space) and the need of preventing overfitting.

### Structure-to-Structure Prediction

Technologies that adopt a simple residue-centric approach, in which secondary structures are predicted independently, often generate inconsistent and unrealistic secondary structure assignment -e.g., isolated alpha-helices. To deal with this problem, a suitable post-processing is usually performed, aimed at improving the prediction accuracy. The post-processing module can be either hand-coded or automatically generated. In the former case, it follows the guidelines of suitable empirical rules, whereas in the latter an architecture typically based on ANNs is devised and trained on the inputs generated by the subsystem responsible for the sequence-to-structure processing. In the implementation of MASSP3 we adhered to the latter approach. In particular, post-processing is performed by a single MLP fed with the sequence-to-structure prediction expressed in terms of *α*, *β*, and *c *propensions. To augment the autocorrelation of the input signal, a suitable "low-pass" filtering is also preliminarily performed by resorting to a suitable gaussian shape (for each class, a value averaged over a window of three residues is evaluated). In so doing, the MLP takes as input the resulting three-dimensional signal on a window of 21 residues and generates three outputs in [0, 1] -to be considered as pseudo-probabilities. Each aminoacid of the given sequence is then labelled with *α*, *β*, or *c *according to a criterion of maximum-likelyhood.

## Results

To perform experiments, the same datasets adopted in [18] have been adopted. The training set (i.e., the TRAIN dataset) contains 1180 sequences, obtained from the PDB database [23] by disregarding proteins with more than 25% of identity with the test set. The test set (i.e., the RS126 dataset) contains 126 non-redundant proteins (no pairs of proteins can be found in the set with more than 25% of similarity over a length of more than 80 residues). Let us point out in advance that MLP inputs are evaluated on a moving window of 15 aminoacids centered on the aminoacid to be predicted (i.e., 7+1+7), each position in the window beng represented by a vector of 21 values in [0, 1] (i.e., 20 for amino acids and one for representing the gap).

Several experiments have been performed, aimed at assessing the impact of different aspects on the behavior of the system: (i) optimization of genetic experts, (ii) input encoding, (iii) experts' specialization technique, and (iv) post-processing technique. Let us stress that post-processing (i.e., structure-to-structure prediction) has been deferred to the final experiment.

The first experiment has been performed using a population of 600 experts with randomly-generated guards. MLPs were supplied with inputs obtained by enforcing a BLAST-based encoding of aminoacids, and were equipped with one hidden layer of 10–25 neurons, depending on the amount of inputs that can be selected by the corresponding guards. Each MLP has been trained using backpropagation and considering only the inputs filtered in by the corresponding guard. Since no selective pressure has been enforced, "good" experts have in fact been counterbalanced by "bad" ones, giving rise to a *Q*_*3*_= 69,1%. This result, considered as a reference, is comparable to the one obtained by a single MLP trained on the whole training set.

The second experiment was aimed at evaluating the impact of the genetic selection on the performance of the system. In this case, the above population has been evolved using covering, single point crossover and mutation operators. The underlying genetic algorithm performed 60 epochs and the final population contained 550 experts (after removing the ones that were unable to match more than 0.1% of the overall inputs used for training). MLPs have been trained using backpropagation and considering only the inputs filtered in by the corresponding guard. Results obtained in this case (71.8%) show an improvement of about 2%, seemingly dependent on the domain knowledge embodied by the most "successful" experts.

In the third experiment, MLPs were trained using backpropagation with a two-tiered policy: in the first 5 epochs each expert was trained on the overall dataset, whereas in the subsequent epochs only the subset of inputs selected by the corresponding guards was used for training. The underlying rationale is that "specialization" may not necessarily occur from scratch; rather, it can originate from a common starting point, obtained by allowing experts to have a global visibility over the input space.

Results (*Q*_*3*_= 73.2) show an improvement of about 1.5%. In the fourth experiment, the position-specific scoring matrices that characterize PSI-BLAST profiles [16] have been adopted to encode MLP inputs. MLPs have been trained using using backpropagation, together with the two-tiered policy described above. Results (*Q*_*3*_= 74.7%) show an improvement of about 1.5%.

Final experiments have been performed by adopting a post-processor, consisting of a single MLP with a moving window 21 aminoacids. To better highlight the correlation among neighbor residues, a preliminary encoding through a lowpass gaussian filter (σ = 0.5) has been performed on the output supplied by the module based on multiple experts. These experiments allowed to reach a *Q*_*3*_= 76.1%. A 7-fold cross-validation performed on the selected training set essentially confirmed this result, with an average *Q*_*3*_= 75.9.

Table 2 summarizes all experimental results described so far, pointing to the accuracy of each specific training strategy or encoding technique. Moreover, Table 3 reports the accuracy of the system and the SOV score [24] for the three secondary structure states and the overall *Q*_*3*_ and SOV score. Let us recall that SOV measures the accuracy of the prediction in terms of secondary structure segments rather than on individual residues.

## Conclusion

In this paper, an approach for predicting protein secondary structures has been presented, which relies on an architecture based on multiple experts. In particular, a population of hybrid experts – embodying a genetic and a neural part – has been devised to perform the given application task. Experimental results, performed on sequences taken from well-known protein databases, point to the validity of the approach. As for the future work, we are investigating the applicability of more powerful features, to be embedded within genetic guards, able to improve their ability of performing context identification. Furthermore, the task of implementing a community of heterogeneous experts is currently being investigated.

## Figures and Tables

**Table 1:** Features used for "soft" partitioning the input space (r denotes the length of the corresponding window)

**Feature**	**Conjecture**
1 Check whether hydrophobic aminoacids occur in the current window (r = 15) according to a clear periodicity (i.e., one every 3–4 residues)	Alpha helices may sometimes fulfill this pattern
2 Check whether the current window (r = 13) contains numerous residues in {A,E,L,M} and few residues in {P,G,Y,S}	Alpha helices are often evidenced by {A,E,L,M} residues, whereas {P,G,Y,S} residues account for their absence
3 Check whether the left side of the current window (r = 13) is mostly hydrophobic and the right part is mostly hydrophilic (and viceversa)	Transmembrane alpha helices may fulfil this feature
4 Check whether, on the average, the current window (r = 11) is positively charged or not	A positive charge might account for alpha helices or beta sheets
5 Check whether, on the average, the current window (r = 11) is negatively charged or not	A negative charge might account for alpha helices or beta sheets
6 Check whether, on the average, the current window (r = 11) is neutral	A neutral charge might account for coils
7 Check whether the current window (r = 11) mostly contains \small" residues	Small residues might account for alpha helices or beta sheets
8 Check whether the current window (r = 11) mostly contains polar residues	Polar residues might account for alpha helices or beta sheets

**Table 2:** Results obtained on the RS126 test set in accordance with the training strategies and encodingtechniques that have been experimented

**Experiment**	**Accuracy**
Random population	69.1
Genetically-selected population	71.8
Improved experts' specialization technique (global + local)	73.2
Using PSI-BLAST profiles	74.7
Using post-processing	76.1

**Table 3:** Detailed results obtained on the RS126 test set using PSI-BLAST profiles and post-processing

	**α**	**β**	**c**	**overall**
**Accuracy**	78.2	63.1	81.3	76.1
**SOV**	77.8	73.2	70.3	71.8
